# Optimization of spray drying process for recovery of onion–stevia leaf hot water extract powder using response surface methodology

**DOI:** 10.1002/fsn3.3207

**Published:** 2023-01-04

**Authors:** Hae‐Il Yang, Kashif Ameer, Young Bae Chung, Sung‐Gi Min, Jong‐Bang Eun

**Affiliations:** ^1^ Department of Integrative Food, Bioscience and Biotechnology Chonnam National University Gwangju Republic of Korea; ^2^ Practical Technology Research Group World Institute of Kimchi Gwangju Republic of Korea; ^3^ Institute of Food Science and Nutrition University of Sargodha Sargodha Pakistan

**Keywords:** onion, powder, RSM, SEM, spray drying, stevia

## Abstract

It is important to optimize the drying process, along with the concentration of drying aid and the inlet air temperature, in order to obtain products with better physicochemical properties. Onion–stevia leaf hot extract powders were prepared using gum arabic (GA) and whey protein concentrate (WPC). Inlet air temperature and carrier concentrations were optimized using response surface methodology. The drying yield of powdered extracts was 14.39–74.32%, L*‐ value was 52.66–66.98, bulk density was 0.36–0.75 (g/cm^3^), moisture content was 2.40–11.57%, water solubility index was 30.32%–97.46%, and mean particle size D[4,3] was 9.13–88.01 (μm). For both GA‐ and WPC‐based powders, optimal inlet air temperatures and carrier concentrations were 148.81 and 144.62°C, and 11.58 and 12.03% (w/v), respectively. GA powders had a higher glass transition temperature (76.49°C) as compared to WPC powders (48.12°C) or maltodextrin as control (55.49°C). Sweetness (5.0/7.0) and overall acceptability (4.3/7.0) scores were higher for GA powders as compared to WPC powders (3.7/7.0 and 3.4/7.0), respectively. Conclusively, GA is a better carrier than whey protein for preparing spray‐dried onion–stevia powder that can be used as a natural sweetener.

## Graphical abstract

Onion–stevia leaf hot extract powders were prepared using gum arabic and whey protein concentrate. Inlet air temperature and carrier concentrations were optimized using response surface methodology. Gum arabic is a better carrier than whey protein for manufacturing spray‐dried onion–stevia powder that can be used as a natural sweetener.

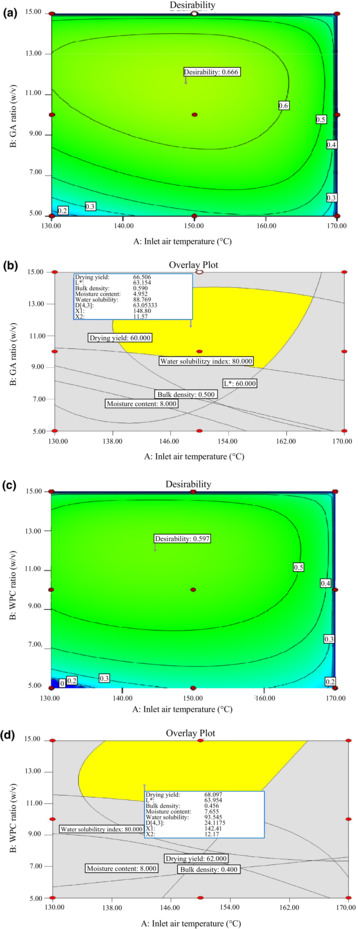

## 1 INTRODUCTION

In the last decades, the consumption of refined sugar has seen rising across the globe. However, refined sugar is absorbed more rapidly into the blood when compared with natural sugars, promoting adiposity and arterial hypertension. In addition, studies on the relationship between refined sugar and cancer have revealed that cancer cells preferentially and readily utilize fructose to support proliferation owing to nucleic acid synthesis. Consumers are looking for healthier alternatives to sugar. Artificial sweeteners, such as acesulfame‐K, aspartame, neotame, saccharin, and sucralose, have attracted attention as substitutes for refined sugar. However, animal studies have reported that some artificial sweeteners are linked to health risks, such as weight gain, brain tumors, and bladder cancer (Ahmad et al., 2020; Ameer, Bae, Jo, Lee, et al., 2017). Natural sweeteners could serve as alternatives to refined sugar and artificial sweeteners without the associated health risks, however, commercial natural sweeteners are usually more expensive than their artificial counterparts (Ameer, Chun, & Kwon, 2017).

In South Korea, postharvest onion production may be subject to annual variability, and overproduction is common; therefore, onions can serve as an inexpensive source of natural sweetener (Sharma, Asnin, et al., 2015). However, Kim et al. (2009) reported that onion hot water extract (OHE) should be improved with respect to its taste and aroma properties to increase the consumers' acceptability. Therefore, in our previous study, onion–stevia leaf hot water extracts (OSHEs) were prepared from onion and stevia leaves without solvent, and were provided higher sensory scores by the panelists for sweetness and overall acceptability. Globally, people in many countries including Korea, Thailand, Vietnam, Brazil, China, Canada, and Japan are consuming stevia leaves and refined extracts as natural sweeteners (Yadav et al., 2011). Sweetening diterpene glycosides consist of stevioside (ST), steviolbioside, dulcoside, rebaudioside‐A (Reb‐A), ‐B, ‐C, ‐D, ‐E, and ‐F. Among all these, ST and Reb‐A are the major sweetening components of stevia leaves accounting for approximately 90% of glycosides (w/w) present in the leaves. On the basis of the total dry weight of stevia leaf, ST and Reb‐A constitute about 5%–18% and 2%–4% of leaf composition, respectively (Ameer, Chun, & Kwon, 2017). Stevia has several health benefits and is generally recognized as safe (GRAS) to use as sugar substitute to sweeten the food products without imparting the negative health effects of refined sugar. Stevia has several reported health benefits, such as reduced risk of cavities and lowered serum glucose levels and caloric intake. Steviosides obtained from stevia are usually employed on an industrial scale as replacers of artificial, intense sweeteners and sucrose in preparing baked goods, dairy products, soft drinks, chewing gums, jams, jellies, mouthwash, and toothpaste. In terms of therapeutic benefits, steviol glycosides exhibit several benefits, such as anticarcinogenic, immunomodulatory, antihyperglycemic, glucagonostatic, antioxidant, and antidiabetic properties. Steviol glycosides have several pharmacological advantages, such as maintenance of serum glucose levels and significant effect on gluconeogenesis (Ameer et al., 2022; Ameer, Bae, Jo, Chung, et al., 2017; Kang et al., 2022). In November 2011, stevia was approved by the European Food Safety Authority (EFSA) to be employed as an additive and noncaloric sweetener across markets of the Europe Union. The acceptable daily intake of about 4 mg/kg body weight was approved by the EFSA, and Food and Drug Administration (FDA) of the United States also granted approval for food additives after granting stevia the GRAS status (Ameer et al., 2022).

Like other fruits and vegetables, onions also contain sugar in their compositions. Onions comprise 4.24% of sugar contents on the basis of wet weight (per 100 g). According to the review of literature of the published reports spanning from 1992 to 2002, the global production of onion has increased to about 25% (approximately 44 million tons). From a time period 2002 to 2011, the Food and Agriculture Organization of United Nations (FAOSTAT) also indicated that onion production escalated up to 85 million tons. Onion comprises of moisture (ranging from 88.6% to 92.8%), fat trace (0.2%), ash (0.6%), and carbohydrates (ranging from 5.2% to 9.1%) (Bogevska et al., 2016; Griffiths et al., 2002; Jiang et al., 2021). The majority of the compositional framework of onion comprises dry matter content having nonstructural carbohydrates, such as fructans, sucrose, fructose, and glucose (Li et al., 2020; Yang et al., 2021). The sugar content of onion is subjected to variability during storage depending on storage temperature, postharvest treatments, and cultivar type. The average sugar content of traditionally stored onion was reported to be 6%. Reducing sugar content of onion at cold room storage was 4.6% (Ameer, Shahbaz, & Kwon, 2017; Bogevska et al., 2016; Sharma, Ko, et al., 2015).

Response surface methodology (RSM), comprising sophisticated mathematical and statistical techniques, is employed for the improvement and optimization of the processes (Ameer, Bae, Jo, Lee, et al., 2017). Spray drying is an excellent method to prepare OSHE powder solution. However, the powders obtained by spray drying are amorphous and susceptible to glass‐transition‐related changes, including stickiness, caking, and collapse, as well as color changes (Bhandari et al., 1997), leading to a low product yield. Most of these problems can be solved by incorporating drying aids. High‐molecular‐weight carbohydrates, such as maltodextrins, decrease the hygroscopicity of powders (Bhandari et al., 1997; Bhandari & Hartel, 2005). Gum arabic (GA), a carbohydrate‐based natural gum obtained by hardening the sap of acacia, has been reported to have higher *T*_*g*_ values than maltodextrin DE10 (MD10) (Collares et al., 2004). Whey protein concentrate (WPC) is the cheapest and most common form of whey protein and a byproduct of cheese production. The concentration of the drying aid and the inlet air temperature exert significant effect on the drying yield, moisture content, hygroscopicity, and solubility during spray drying (Bakar et al., 2013; Chen et al., 2014). Therefore, it is important to optimize the drying process, along with the concentration of drying aid and the inlet air temperature, in order to obtain products with better physicochemical properties.

The study aimed to optimize the inlet air temperature and the concentration of the drying aid, when preparing OSHEP with GA (OSHEP‐GA) and WPC (OSHEP‐WPC), for obtaining better drying yield, color (L*), bulk density, moisture content, water solubility index, and mean particle size, using RSM. In addition, optimized OSHEP‐GA and OSHEP‐WPC were compared with OSHEP produced with maltodextrin 20 DE (OSHEP‐MD), as a positive control, using scanning electron microscopy (SEM), differential scanning calorimetry (DSC), and sensory evaluation.

## 2 MATERIAL AND METHODS

### 2.1 Materials

Onion (*Allium cepa* L.) was supplied from a local farm in Muan, South Korea. The tops and bottoms of onion bulbs were removed, washed with water, and divided into eight parts. Dried leaves of *Stevia rebaudiana* were purchased from a local farm in Pyeongtaek, South Korea. Gum arabic (GA) was obtained from ES Food Ltd. (Gunpo, South Korea). Whey protein concentrate (WPC) was procured from Shin Bi International (Pocheon, South Korea).

### 2.2 Experimental design

A Box–Behnken design (BBD) was used for optimization when one process and two formulation conditions were being investigated, such as inlet air temperature with GA and WPC concentrations. The measured responses were drying yield, L* values, bulk density, moisture content, water solubility index, and mean particle size. The experimental layout based on Box–Behnken design (BBD) configuration is shown in Table 1. The Design‐Expert software (v.8.0.6, Stat‐Ease, Inc., Minneapolis, MN, USA) was used to design the experiments and analyze the experimental data. The experimental data were adjusted to a quadratic model to express the response variables as a function of the independent variables using the following equation:

(1) $$Y=\beta_{0}+\sum_{i=1}^{4}\beta_{i}X_{i}+\sum_{i=1}^{4}\beta_{\mathrm{ii}}X_{i}^{2}+\sum_{i=1}^{3}\sum_{j=1}^{4}\beta_{\mathrm{ij}}X_{i}X_{j}$$

where *Y* represents response variable, $\beta_{0}$, $\beta_{i},\beta_{\mathrm{ii}},\&\beta_{\mathrm{ij}}$ denote regression coefficients and $X_{i},X_{j,}\&X_{\mathrm{ij}}$ were indicative of coded independent variables.

**TABLE 1:** The experimental data for the response surface analysis of the effect of processing conditions on the quality of onion–stevia leaf hot extract powder (OSHEP) with different carriers.

OSHEP prepared by gum arabic as carrier agent								
Run	Factor 1: A: Inlet air temperature (°C)	Factor 2: B: Carrier (GA) concentration (w/v)	R1: Drying yield (%)	R2: L* value	R3: Bulk density (g/cm^3^)	R4: Moisture content (%)	R5: Water solubility index (%)	R6: D[4,3] (μm)
1	150	15	54.28 ± 5.24^a^	63.15 ± 4.61	0.75 ± 0.01	3.22 ± 1.31	87.08 ± 2.10	77.54 ± 4.11
2	150	10	66.73 ± 4.97	62.62 ± 3.96	0.57 ± 0.03	6.04 ± 1.91	85.54 ± 4.13	83.74 ± 5.29
3	130	15	46.01 ± 3.89	62.76 ± 5.28	0.57 ± 0.09	3.67 ± 1.12	83.60 ± 5.18	40.66 ± 3.61
4	130	5	14.39 ± 2.24	58.37 ± 5.44	0.40 ± 0.05	10.12 ± 2.14	30.32 ± 2.14	9.13 ± 1.19
5	130	10	54.90 ± 5.53	62.12 ± 3.67	0.50 ± 0.01	6.40 ± 1.17	80.63 ± 3.63	43.96 ± 4.22
6	170	10	65.14 ± 4.89	55.99 ± 5.32	0.61 ± 0.03	4.37 ± 1.16	87.76 ± 4.71	62.74 ± 5.29
7	170	15	50.26 ± 4.41	59.40 ± 4.25	0.67 ± 0.02	2.40 ± 0.09	98.65 ± 5.91	88.00 ± 6.38
8	170	5	63.27 ± 5.43	52.66 ± 3.96	0.45 ± 0.04	7.03 ± 1.17	48.10 ± 4.32	84.20 ± 5.21
9	150	5	48.54 ± 3.41	59.16 ± 4.11	0.53 ± 0.06	8.56 ± 1.12	34.60 ± 3.17	70.04 ± 4.37
10	150	10	66.81 ± 4.49	62.63 ± 3.32	0.56 ± 0.04	5.99 ± 1.16	85.97 ± 5.26	80.64 ± 4.29
11	150	10	66.68 ± 5.73	62.73 ± 4.61	0.58 ± 0.05	6.10 ± 1.14	85.11 ± 4.31	86.84 ± 5.71
**OSHEP prepared by whey protein concentrate as carrier agent**								
**Run**	**Factor 1: A:** **Inlet air temperature (°C)**	**Factor 2: B:** **Carrier (GA) concentration (w/v)**	**R1: Drying yield (%)**	R2: L* value	**R3: Bulk density (g/cm** ^**3**^ **)**	**R4: Moisture content (%)**	**R5: Water solubility index (%)**	**R6: D[4,3] (μm)**
1	130	5	41.56 ± 4.12	62.20 ± 4.78	0.42 ± 0.02	11.57 ± 1.43	75.62 ± 3.43	22.12 ± 2.39
2	170	10	74.32 ± 5.28	53.66 ± 3.19	0.42 ± 0.01	7.30 ± 1.35	97.27 ± 4.32	33.84 ± 3.15
3	170	15	69.68 ± 4.67	57.16 ± 4.01	0.48 ± 0.04	6.54 ± 1.29	97.46 ± 5.51	32.26 ± 2.32
4	150	5	45.93 ± 4.78	58.50 ± 4.67	0.36 ± 0.01	9.03 ± 1.51	77.21 ± 4.18	19.38 ± 2.46
5	130	10	66.56 ± 5.81	65.56 ± 4.91	0.44 ± 0.03	7.75 ± 1.35	85.14 ± 4.35	21.94 ± 2.78
6	150	15	66.66 ± 6.03	65.88 ± 4.18	0.50 ± 0.06	8.46 ± 1.76	96.22 ± 5.79	25.60 ± 2.83
7	170	5	67.39 ± 5.14	55.20 ± 3.62	0.38 ± 0.04	8.48 ± 1.89	81.71 ± 4.61	21.32 ± 2.12
8	150	10	69.77 ± 4.27	64.87 ± 4.29	0.42 ± 0.03	8.43 ± 1.91	94.90 ± 5.97	22.84 ± 1.91
9	130	15	63.88 ± 4.19	66.98 ± 4.16	0.47 ± 0.06	6.08 ± 1.54	86.46 ± 4.38	22.36 ± 2.08
10	150	10	69.69 ± 4.09	64.62 ± 4.11	0.41 ± 0.02	8.25 ± 1.43	99.47 ± 5.92	23.10 ± 2.05
11	150	10	69.64 ± 4.06	65.17 ± 3.97	0.43 ± 0.06	8.61 ± 1.39	94.31 ± 5.38	22.58 ± 2.07
**Glass‐transition temperature (*Tg*) of OSHEPs produced with different carriers**								
	**Maltodextrin 20 DE**	**Gum arabic**	**Whey protein concentrate**					
*T*_*g*_ (°C)	55.49 ± 2.49	76.49 ± 1.06	48.12 ± 1.03					

*Note*: *T*_*g*_ is measured by DSC.

^a^ Values are mean ± standard deviation (*n* = 3).

In addition, the simultaneous optimization of different responses was carried out through a numerical enhancement procedure. The objectives for each factor and response were selected and the response of each trial was analyzed to predict the interactive effects of different parameters on the physicochemical properties of OSHEP and to define the optimal conditions (Table 2).

**TABLE 2:** Criteria and outputs for the numerical optimization of the responses for each onion–stevia leaf hot extract powder (OSHEP) with different carriers.

Name	Goal	Lower limit	Upper limit	Lower weight	Upper weight	Importance
A: Inlet air temperature	Minimize	130	170	1	1	3
B: GA concentration	Minimize	5	15	1	1	3
Drying yield	Maximize	14.39	66.74	1	1	3
L*	Maximize	52.66	63.15	1	1	3
Bulk density	Maximize	0.40	0.75	1	1	3
Moisture content	Minimize	2.40	10.12	1	1	3
Water solubility index	Maximize	30.32	98.65	1	1	3
D[4,3]	Maximize	9.13	88.00	1	1	3
A: Inlet air temperature	Minimize	130	170	1	1	3
B: WPC concentration	Minimize	5	15	1	1	3
Drying yield	Maximize	41.56	74.32	1	1	3
L*	Maximize	53.63	66.98	1	1	3
Bulk density	Maximize	0.36	0.50	1	1	3
Moisture content	Minimize	6.08	11.57	1	1	3
Water solubility index	Maximize	75.62	97.46	1	1	3

Significance level of powder responses for different carriers by RSM						
P > F						
	Drying yield (%)	L*	Bulk density	Moisture content (%)	Water solubility index (%)	D[4,3] (μm)
Model	0.0331	0.0087	0.0097	<0.0001	0.0025	0.0404
A: Inlet air temperature	0.0190	0.0044	0.1144	0.0003	0.0232	0.0176
B: GA concentration	0.1733	0.0045	0.0050	<0.0001	0.0005	0.3633
AB	0.0281	0.2386	‐	0.0258	0.7432	‐
A^2^	0.1529	0.0118	‐	‐	0.4308	‐
B^2^	0.0265	0.1726	‐	‐	0.0044	‐

	Drying yield (%)	L*	Bulk density	Moisture content (%)	Water solubility index (%)	D[4,3] (μm)
Model	0.0366	0.0048	0.0024	0.0576	0.0160	0.0596
A: Inlet air temperature	0.0299	0.0027	0.2862	0.2693	0.0159	0.0447
B: WPC concentration	0.0206	0.0531	0.0009	0.0234	0.0045	0.0784
AB	0.0935	–	–	0.1402	0.3837	0.1574
A^2^	0.3616	–	–	–	0.2964	–
B^2^	0.0325	–	–	–	0.0301	–

Subsequently, OSHEP‐GA and OSHEP‐WPC were prepared under optimal conditions. As a control, OSHEP was produced with MD (OSHEP‐MD) using the same ratio as that of carbohydrate‐based GA, and the products were compared by scanning electron microscopy (SEM), differential scanning calorimetry (DSC), and sensory evaluation.

### 2.3 Onion–stevia leaf hot extract (OSHE) preparation

Based on our previous study, OSHE was prepared by the following process as per the reported method of Yang et al. (2021). Onion and stevia (onion:stevia ratio = 100:1, w/w) were heated at 115°C, for 4 h, at a pressure of 1.2 kgf/cm^2^, using a retort (STERI‐ACE PRS‐60‐1, Kyunghan Co., Ltd, Gyeongsan, South Korea). Then, the heated onion and stevia were juiced at 200 kgf/cm^2^ with a 60‐L hydraulic turbine (Sungchang Co., Ltd, Namyangju, South Korea) and filtered using a 11μm Whatman filter paper No. 1 (Whatman International Ltd., Maidstone, England) prior to spray drying. The total soluble solid content of the prepared OSHE was 5.7 ± 0.1 °Brix determined using a digital refractometer (HI 96801, Hanna Instruments Inc., Woonsocket, USA). The storage of prepared OSHE was carried out at −20°C in 50‐ml tube until further use.

### 2.4 Spray drying

A pilot‐scale spray dryer (MH‐8, Mehyun Engineering, Seoul, South Korea) was standardized at a feed rate of 12 ml/min and air pressure of 7.55 kgf/cm^2^. The suspension was provided to the main chamber by means of peristaltic pump and nozzle having a diameter of 0.5 mm and the feed flow rate was changed through the maneuvering of pump rotation speed. The drying inlet air temperature and carrier‐to‐OSHE ratio (w/v) were varied based on BBD‐based experimental design (Table 1). The ranges of inlet air temperature (130–170°C) and carrier‐to‐OSHE ratio (5%–15%, w/v) were fixed based on the outcomes of pretrials and references (Bazaria & Kumar, 2016). The outlet temperature was adjusted to half of that of the temperature employed for inlet air. The samples were taken from the product collection vessel. An insulated glass bottle was utilized for powder collection which was connected to the cyclone end after the completion of drying process. The powder was subjected to packing in the polyethylene pouches and stored in the desiccator at 25°C in the presence of silica gel till further analysis.

### 2.5 Physicochemical properties

#### 2.5.1 Drying yield

Drying yield was determined by dividing the mass of the powder collected in the product collector by the total solids in the feed in the main chamber of a spray dryer (da Silva Bastos et al., 2012). The total solid content represents the mass of soluble and insoluble solids present in the OSHEs, including the carrier (GA or WPC) added to the formulation. The total soluble solid content of the OSHEs was gravimetrically measured by drying in an oven (FO‐600 M, Jeio Tech, Seoul, South Korea) at 105°C for 24 h.

#### 2.5.2 Degree of lightness (L*) measurement

The color of OSHE (5 ml) was determined using a colorimeter (CR‐400, Minolta, Tokyo, Japan). Calibration was performed on a white tile prior to sample analysis (L* = 86.90, a* = 0.3170, and b* = 0.3240). The results were expressed as the degree of lightness values (L*).

#### 2.5.3 Bulk density

Bulk density was calculated as per the described method of AOAC method No. 110–145. 127 (AOAC, 2000). The bulk density of the powder was measured by weighing 2 g of each sample and placing it in a graduated cylinder (10 ml). The cylinder was lightly tapped by hand and the bulk density was calculated as the ratio of the mass of powder contained in the cylinder to the volume occupied (Mahdavi et al., 2016).

$$\text{Bulk density}\;\left(g/\mathrm{ml}\right)=\frac{\text{Mass of powder}}{\text{Volume}}$$

#### 2.5.4 Moisture content

Moisture content was calculated as per the described method of AOAC method No. 925.10 (AOAC, 2000). The moisture content of OSHEP (2 g) was determined by drying it in an oven at 105°C to a constant weight (Rasul et al., 1999).

#### 2.5.5 Water solubility index (WSI)

The WSI was determined as described by Anderson (1969) with modifications. To determine WSI, 2.5 g of powder was suspended in 30 ml of distilled water at ambient temperature in a tarred centrifuge tube. The suspension was stirred in a vortex mixer for 1 min, placed in a water bath (JSSB‐50 T, 3.2 KW, 14.5 A 1P, JS Research Inc., Gongju, South Korea) at 37°C for 30 min, and then spun in a centrifuge (Union32R Plus, Hanil Scientific, Seoul, South Korea) at 3500 × *g* for 20 min. The liquid supernatant was poured into a preweighed dish and dried at 105°C to a constant weight. WSI was calculated by the following formula:

(2) $$\mathrm{WSI}\;\left(\%\right)=\frac{\text{Dried supernatant weight}\;\left(g\right)}{\text{Initial sample weight}\;\left(g\right)}\times 100$$

### 2.6 Particle size distribution

Operational conditions may exert a significant influence on the particle size distribution of powdered products and it is one of the most influential factors for assessing the physicochemical properties of spray‐dried powders. The particle size distribution in the spray‐dried powders was determined by dispersing the particles in distilled water and using the laser light‐scattering method with a particle size analyzer (Mastersizer 3000, Malvern Instruments Ltd., Worcs., UK) with a dry powder feed unit. The maximum flow rate was adjusted for the airflow in conjunction with 20% feed rate as the maximum value. In order to improve the particle size distributions for spray‐dried powdered products, fine‐particle mode was employed. For analytical purposes, size range from 0.1 to 1000 μm was used. The average particle size and the specific surface area were reported, as explained above. The mean particle size was expressed as D [4,3].

### 2.7 Scanning electron microscopy (SEM)

The microstructure of the OSHEPs was observed by a scanning electron microscope (JSM‐IT300LV, JEOL, Tokyo, Japan). Preparation of spray‐dried samples for SEM imaging was carried out through placement of sample over carbon tape on an aluminum stab. Powders were sputter coated with platinum, and examined by a scanning electron microscope operated at 10 kV at 200× and 700× magnifications.

### 2.8 Sensory evaluation

The sensory evaluation was carried out by 50 untrained panelists who were students of the Department of Food Science and Technology, Chonnam National University (Gwangju, South Korea). The sensory attributes of each OSHEP sample were scored by the panelists, using a 7‐point hedonic scale (ranging from 1 to 7, with 1 = extremely weak and 7 = extremely strong) for color, solubility, sweetness, saltiness, sourness, onion taste, and milky taste. Overall acceptance was also scored on a 1–7 scale, with 1 = extremely disliked and 7 = extremely well‐liked. This study was approved by the Institutional Review Board (IRB) of Chonnam National University (IRB No. 1040198‐191,210‐HR‐123‐02), and received permission for informed consent and approved supervision for the sensory evaluation analysis. All participants cleansed their palates by consuming water and unsalted crackers and using expectorant cups during the intervals between samples (Choi et al., 2014).

### 2.9 Differential scanning calorimetry (DSC)

A differential scanning calorimeter (DSC823e, Mettler Toledo AG, Schwerzenbach, Switzerland) was used to determine the glass transition temperature (*T*_*g*_) of all OSHEPs. The employed purge gas was dry nitrogen which was purged at a rate of 20 ml/min. Although onset and end values for *T*_*g*_ samples were calculated for each DSC thermogram, only the *T*_*g*_ values determined at half the extrapolated change in specific heat (ΔCp), between the glassy state and the rubbery state, were reported in this study. Indium and zinc were used for calibration of temperature and heat flow. An empty aluminum pan was used as a reference. Ten milligrams of sample were scanned in a hermetically sealed 50‐μl DSC aluminum pan. Thermal scanning was carried out in the following order unless described otherwise: (1) isothermal at −20°C for 1 min; (2) heat scanning from −20°C to temperature just over predetermined apparent *T*_*g*_ at 10 °C/min; (3) cooling rapidly −20°C at 50°C/min; and (4) heat scanning from −20 to 200°C at 10°C/min. A heating rate of 10°C/min was chosen as a standard. The second scan of each sample was used to reduce the enthalpy relaxation of the amorphous powder which appears in the first scan, thereby enhancing the accuracy of *T*_*g*_ measurement in our differential scanning calorimetry thermograms. The transfer of samples from the container to the DSC pan was done in a sealed “dry box” containing silica gel, with regular N_2_ flushing, to avoid moisture absorption by the sample (Shrestha et al., 2007).

### 2.10 Statistical analysis

All experiments were conducted in triplicate and presented as mean ± standard deviation (S.D.). Significance of the data obtained was analyzed by one‐way analysis of variance (ANOVA), whereas differences between the means were compared by Duncan's multiple‐range test using SPSS version 18.0 (SPSS Institute, Chicago, IL, USA) at a significance level of *p* < 0.05.

## 3 RESULT AND DISCUSSIONS

### 3.1 Physicochemical properties

#### 3.1.1 Drying yield

**FIGURE 1:**
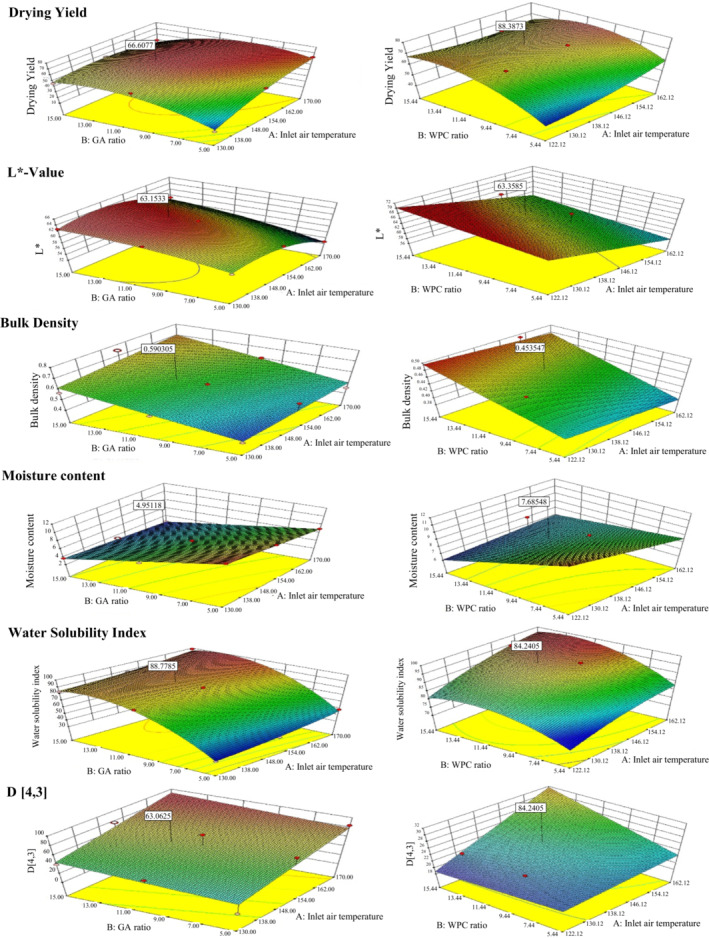
Response surface and contour plot for determining the effect of cross‐interaction between A (inlet air temperature) and B (carrier ratio) on the physicochemical properties of OSHEP.

The effect of varying the inlet air temperature and the concentration of the carrier on drying yield are shown in Figure 1. The drying yield increased with inlet air temperature, up to 170°C, and then decreased slightly. This could be explained by the fact that temperatures lower than 150°C may decrease the probability of the drying particles hitting the wall of the drying chamber, but at temperatures higher than 150°C, the carbohydrates (in GA) might be fused in sweetener‐rich OSHEP, resulting in decreased drying yield (Fazaeli et al., 2012). Powder prepared with 10% carrier exhibited the best drying yield. An increase in product yield with increasing concentration of carrier has been reported for tamarind juice by Cynthia et al. (2015). The main reason for this is that higher carrier concentrations result in higher glass‐transition temperatures of the mixture. A similar result was also reported for sapodilla juice, at 30% maltodextrin by Chong and Wong (2015).

Overall, the drying yields were significantly higher (*p* < 0.05) for OSHEP‐WPC than for OSHEP‐GA. The drying yield of OSHEP‐WPC may be higher than that of OSHEP‐GA in sugar‐rich materials because of the lower surface tension of the protein‐enriched feed (Samborska et al., 2015). Vivek et al. (2021) carried out research work on the optimization of sohiong fruit powder obtained by spray drying and the authors reported that drying yield exhibited significant rise with corresponding increases in the feed rate and maltodextrin concentration. The highest yield of fruit powder obtained was 65.49% at an inlet feed rate of 180 ml/h, 25% maltodextrin concentration, and inlet temperature of 140°C.

The predicted polynomial model was as follows:

$$\begin{array}{cc} \text{Drying yield}\;\left(\text{OSHEP}-\mathrm{GA}\right)=\; & +67.28+10.56^{*}A+4.06^{*}B\;-11.16^{*}A^{*}B\\ & -7.53^{*}A^{2}-16.14^{*}B^{2} \end{array}$$

$$\begin{array}{cc} \text{Drying yield}\;\left(\text{OSHEP}-\mathrm{WPC}\right)=\; & +68.11+6.56^{*}A+7.56^{*}B-5.01^{*}A^{*}B\\ & +3.13^{*}A^{2}-11.01^{*}B^{2} \end{array}$$

where A referred to inlet air temperature while B was indicative of carrier concentration.

#### 3.1.2 Degree of lightness (L*‐value)

Figure 1 shows the effects of different carrier concentrations and inlet air temperatures on the L*‐values of OSHEP. The L* value showed a negative correlation with inlet air temperature and a positive correlation with carrier concentration. The color of the final product of spray‐dried powder usually depends on the color of the carrier. It has been reported that blackberry juice (*Rubus fruticosus*) powder, produced with WPC as a carrier, had higher L* and b* values, and lower a*, than that produced with GA; this is in line with our findings (Díaz et al., 2015). However, at an inlet air temperature 170°C, OSHEP‐GA had a higher L* value than OSHEP‐WPC. It could be that protein‐based WPC had been denatured at that temperature. Likewise, the L* value decreased and the browning index increased as the inlet air temperature increased. It could be that an increase in inlet air temperature caused an increase in nonenzymatic browning of OSHEP (Chen et al., 2014). Vivek et al. (2021) carried out research work on optimization of sohiong fruit powder obtained by spray drying and authors reported that the degree of whiteness of sohiong fruit powder exhibited significant increase with corresponding rises in the inlet feed rate, inlet temperature, and maltodextrin concentration.

The predicted polynomial model was as follows:

$$\begin{array}{cc} L^{*}\;\left(\text{OSHEP}-\mathrm{GA}\right)=\; & \;+62.33-2.53^{*}A+2.52^{*}B+0.59^{*}A^{*}B\\ & \;-3.11^{*}A^{2}-1.01^{*}B^{2} \end{array}$$

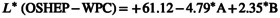




#### 3.1.3 Bulk density

The bulk density of OSHEP was affected by carrier concentration and inlet air temperatures (Figure 1). The bulk density increased with increasing carrier concentration and was the highest at an inlet air temperature of 150°C. The increase in the total soluble solid content of the feed (due to the addition of a carrier) results in puffing or ballooning, and eventual cracking of the particles, causing an increase in bulk density. The increase in bulk density at temperatures below 150°C could be explained by the formation of a hard crust on the surface of the particles, which prevents diffusion and evaporation of moisture (Goula & Adamopoulos, 2004). However, the bulk density at 150–170°C inlet air temperature decreased because of a faster evaporation rate at those temperatures than at lower temperatures, which may cause inflation, forming more porous or fragmented particles. Similarly, corresponding to our results, Nadeem et al. (2011) reported that spray‐dried powder of a water extract of mountain tea (*Sideritis stricta*) exhibited the highest bulk density at an inlet air temperature of 155°C, and showed increasing bulk density with an increasing concentration of the carrier.

In addition, the bulk density was significantly (*p* < 0.05) higher in OSHEP‐GA than in OSHEP‐WPC. Díaz et al. (2015) reported that the bulk densities of powders manufactured with a carbohydrate‐based carrier were higher than those with a protein‐based carrier, and this was attributed to the molecular weight of the carrier. This was in agreement with the findings of this study. Bulk density of the spray‐dried Cempedak (*Artocarpus integer*) powder was studied by Pui et al. (2021) and the authors reported that bulk density was significantly affected by maltodextrin concentration and inlet air temperature. Inlet air temperature exhibited negative correlation with the bulk density and it may be implied that powder packing requires smaller volume and accrued reduced transportation cost. Furthermore, maltodextrin concentration also negatively affected bulk density as maltodextrin concentration may lead to increased trapping of air spaces in the particulate matter (Pui et al., 2021).

The predicted polynomial model was as follows:

$$\text{Bulk density}\;\left(\text{OSHEP}-\mathrm{GA}\right)=+0.56+0.043^{*}A+0.10^{*}B.$$

$$\text{Bulk density}\;\left(\text{OSHEP}-\mathrm{WPC}\right)=+0.43–9.084\;E-003^{*}A+0.048^{*}B.$$

#### 3.1.4 Moisture content

Figure 1 shows the moisture content of OSHEP with different carrier concentrations and inlet air temperatures. Moisture content decreased with increasing carrier concentration and inlet air temperature. Higher inlet air temperatures provided more energy to the droplets and increased heat transfer, which decreased moisture content. Moreover, increased carrier concentration reduced the amount of free water for evaporation, resulting in a decrease in moisture content.

Additionally, the moisture content of OSHEP‐WPC was higher than that of OSHEP‐GA. This could be explained by the different chemical structures of the carriers because proteins (WPC) are more hydrophilic than polysaccharides (GA) (Du et al., 2014). Vivek et al. (2021) carried out research work on optimization of sohiong fruit powder obtained by spray drying and the authors reported that moisture content is one of the most influential parameters of spray‐dried powdered products. The moisture content of spray‐dried sohiong fruit powder ranged 3.15%–4.51%. Fruit powders with reduced moisture content exhibit longer shelf life and excellent storage stability.

The predicted polynomial model was as follows:

$$\text{Moisture content}\;\left(\text{OSHEP}-\mathrm{GA}\right)=+5.76–1.07^{*}A-2.74^{*}B+0.46^{*}A^{*}B.$$

$$\text{Moisture content}\;\left(\text{OSHEP}-\mathrm{WPC}\right)=+8.18–0.51^{*}A-1.33^{*}B+0.89^{*}A^{*}B.$$

#### 3.1.5 Water solubility index (WSI)

The effects of different inlet air temperatures and carrier concentrations on WSI are shown in Figure 1. WSI increased with the concentration of carrier [from 5% to 10% (w/v)] and with inlet air temperature. This could be explained by the superior solubility of both GA and WPC. However, there was no significant difference (*p* > 0.05) when the carrier concentration further increased from 10 to 15% (w/v). Nadeem et al. (2011) and Muzaffar and Kumar (2016) reported a slight increase in solubility when using very high levels of GA with soya protein isolate. Similarly, the solubility of OSHEP‐GA and OSHEP‐WPC also increased slightly when increasing the carrier concentration from 10% to 15% (w/v), but this was not significant. Studies related to pitaya extract powder (Bakar et al., 2013) and mountain tea powder (Nadeem et al., 2011) have revealed that a higher inlet temperature results in higher solubility. The increase in solubility with an increase in inlet air temperature is due to its effect on residual moisture content (Nadeem et al., 2011). In another report by Jafari et al. (2017), the authors have elucidated on influence of spray drying parameters on water solubility index of powdered product, such as pomegranate juice powder. It was concluded by the authors that WSI is one of the crucial parameters which plays decisive role in determining the aqueous behavior and reconstitution ability of the powder. Gradually increasing manufacture in temperature may cause a slight increase in WSI possibly owing to low density as a result of high temperature larger particles exhibit high sinking ability in water as compared to smaller particles which float on the water surface.

The WSI of OSHEP‐WPC was higher than that of OSHEP‐GA, and this could be attributed to the fact that the viscosity decreased, similar to that in a previous study where whey protein powder was added to liquid yogurt and buttermilk (Patocka et al., 2006). Subsequently, the particle size of the final product was reduced because of the lower viscosity, and lower particle size caused higher solubility because of the increase in surface area of the powder contacting the water (Goula & Adamopoulos, 2004).

The predicted polynomial model was as follows:

$$\mathrm{WSI}\;\left(\text{OSHEP}-\mathrm{GA}\right)=+83.02+6.66^{*}A+26.05^{*}B-0.68^{*}A^{*}B+2.44^{*}A^{2}-20.92^{*}B^{2}.$$

$$\mathrm{WSI}\;\left(\text{OSHEP}-\mathrm{WPC}\right)=+93.86+4.87^{*}A+7.60^{*}B+1.23^{*}A^{*}B-2.15^{*}A^{2}-6.64^{*}B^{2}.$$

### 3.2 Particle size distribution

Figure 1 presents the particle size distribution of OSHEPs prepared using 150°C inlet air temperature and 10% carrier concentration, for GA and WPC. The particles exhibited a very broad range in size, with diameters varying from 0.1 to 859.0 μm, approximately. The size showed a bimodal distribution, with two distinct peaks (OSHEP‐GA: approximately 1.0 μm and 100 μm; OSHEP‐WPC: approximately 1.0 μm and 40 μm). Each peak represented a predominant size and was significantly different for OSHEPs produced with different carriers.

**FIGURE 2:**
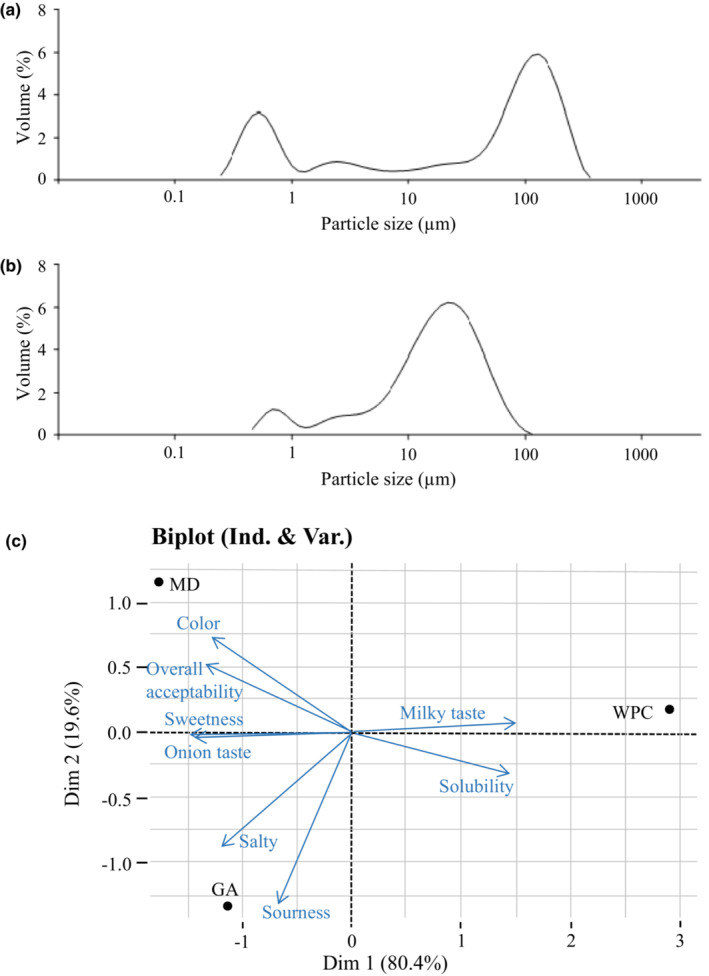
Particle size distributions of onion–stevia leaf hot extract powder (OSHEP) at 150°C inlet air temperature and a 10% concentration of different carriers (w/v). (a) Gum arabic (GA), (b) whey protein concentrate (WPC), and (c) principal component analysis (PCA) for sensory evaluation of OSHEPs produced with different carriers. MD: OSHEP‐MD; GA: OSHEP‐GA; WPC: OSHEP‐WPC.

In addition, the mean particle diameters varied from 9.13 to 88.00 μm (Figure 2 a‐b). The mean particle diameter of OSHEP‐WPC (23.61 μm) was lower than that of OSHEP‐GA (60.82 μm). This may be related to the high solubility of OSHEP‐WPC because this causes the viscosity to be low, and the viscosity of the feed influences the droplets formed during atomization, leading to a smaller particle size in the final product (Goula & Adamopoulos, 2004). Generally, spray‐dried powders have a small particle size (<50 μm), which indicates poor properties associated with handling and reconstitution (Gong et al., 2007). However, the mean particle diameter of OSHEP‐GA, prepared above 150°C, was >50 μm, so the handling and reconstitution properties of this powder are expected to be better than those prepared under other conditions. This result may be related to swelling and prevention of shrinkage observed with increased drying temperature (Islam Shishir et al., 2016).

The predicted polynomial model was as follows:

$$D\left[4,3\right]\;\left(\text{OSHEP}-\mathrm{GA}\right)=+62.22+23.53^{*}A+7.14^{*}B.$$

$$D\left[4,3\right]\;\left(\text{OSHEP}-\mathrm{WPC}\right)=+24.63+3.50^{*}A+2.90^{*}B+2.67^{*}A^{*}B.$$

### 3.3 Optimized spray‐dried OSHEP production

The conditions for optimization of spray‐dried OSHEP production can be seen in Table 1. Multiple‐response optimization suggests that the optimal inlet air temperature and proportion of carrier for producing the best spray‐dried products were achieved for OSHEP‐GA at 148.81°C with 11.58% carrier, and for OSHEP‐WPC at 144.62°C with 12.03% carrier.

**FIGURE 3:**
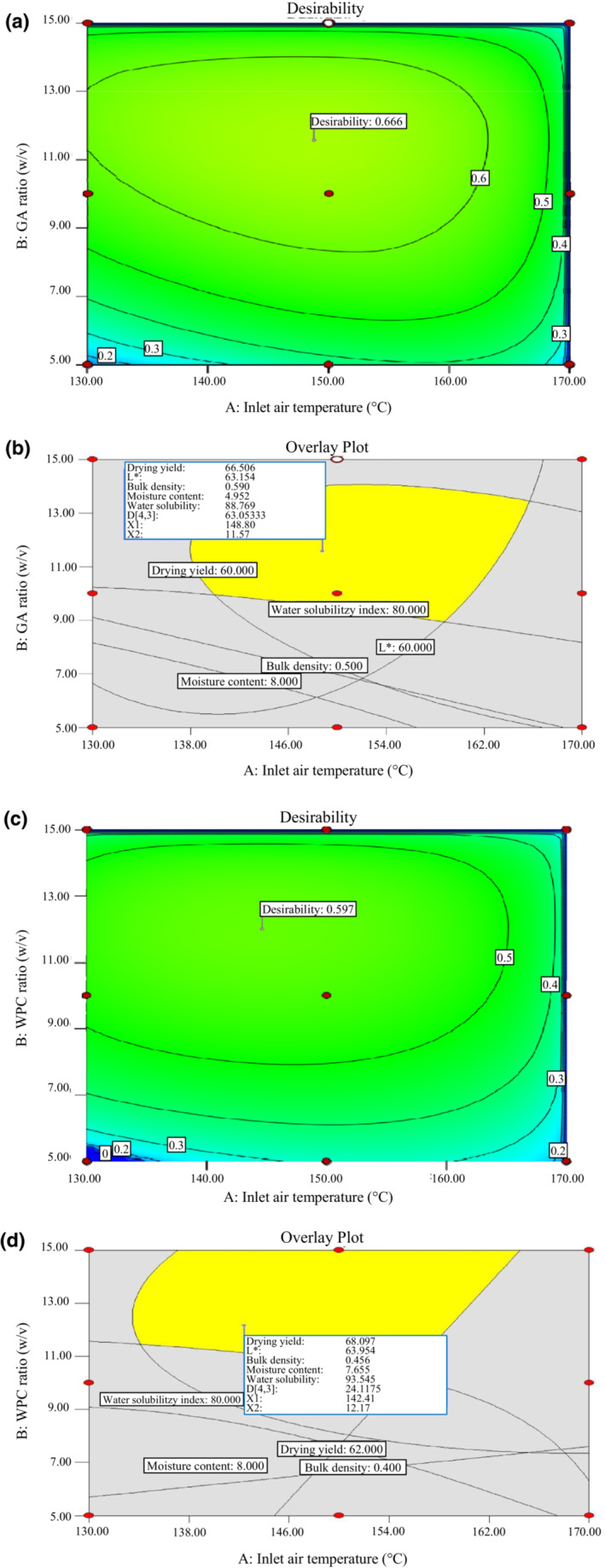
Overlay and desirability charts generated by the constraints for GA (a and b) and WPC (c and d)

At these optimal conditions, the following were the predicted responses for OSHEP‐GA: drying yield = 66.51%, L* = 63.15, bulk density = 0.59, moisture content = 4.95%, water solubility index = 88.78%, and D[4,3] = 63.08 μm. For OSHEP‐WPC, the following were the predicted responses: drying yield = 68.37%, L* = 63.36, bulk density = 0.45, moisture content = 7.68%, water solubility index = 94.25%, and D[4,3] = 24.57 μm. The significance level of powder responses, for different carriers (by RSM), is shown in Table 1. The overlay and desirability charts generated by the constraints are shown in Figure 3a–d.

### 3.4 Scanning electron microscopy (SEM)

**FIGURE 4:**
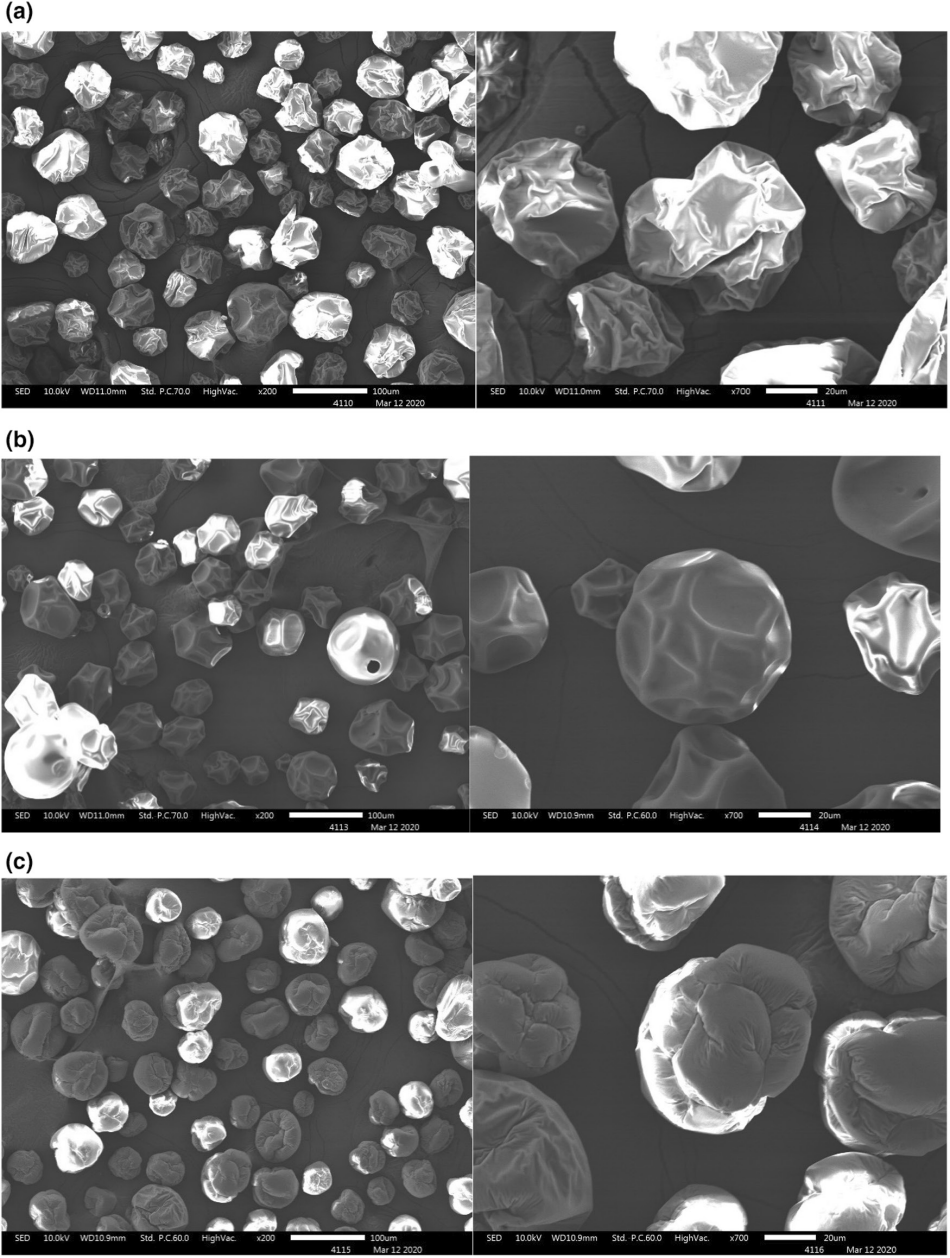
The scanning electron microscopy (SEM) image of OSHEPs produced using optimal inlet air temperature and carriers, (a) GA, (b) WPC, and (c) maltodextrin 20DE^1^. ^1^Maltodextrin follows the optimum conditions of GA.

Figure 4 shows the microstructure of OSHEPs produced with different carriers at optimum conditions. The OSHEP‐GA was wrinkled and had surface dents, without holes, and showed individually stable particles that did not agglomerate with each other. Dents can be formed by the shrinkage of the particles during the drying and cooling of spray‐dried powders and can have an adverse effect on their flow proprieties, but they do not affect the storage stability (Finotelli & Rocha‐Leão, 2005). In many cases, these initially spherical droplets form particles with irregular surfaces (folds) due to the formation of vacuoles, dents, and depressions internally and by external fracture. According to Sheu and Rosenberg (1998), powders spray dried with polysaccharide carriers exhibited notable surface indentations, attributed to the effects of wall composition, atomization, and drying parameters, uneven shrinkage at early stages of drying, and to the effect of a surface tension‐driven viscous flow. The thermal expansion of air or water vapor inside the drying particles can smooth out dents, to a varying extent. The OSHEP‐MD showed wrinkles in a manner similar to OSHEP‐GA.

However, the particles of OSHEP‐WPC showed partial disruption observed in the crust of a few individual particles, suggesting that hollow spheres were formed during spray drying. According to Mimouni et al. (2010), the external layer of the microstructure of milk protein concentrate powder has the appearance of a fragile framework that precariously supports the spherical structure of the particles on the verge of collapsing and fully dispersing. Our observations suggest that dissolution of the powder particles occurred by gradual erosion from both external and internal surfaces, exposed by breaches in the outer layer.

In conclusion, considering the holes observed in particles of OSHEP‐WPC and the similarity of OSHEP‐GA to the microstructure of OSHEP‐MD, it was concluded that GA is a better carrier than WPC for manufacturing OSHEP.

### 3.5 Differential scanning calorimetry (DSC)

The DSC technique was used to obtain heat flow versus temperature thermograms. *T*_*g*_ is observed as an endothermic stepwise change in the heat flow. Table 1 shows the *T*_*g*_ of OSHEPs produced with different carriers at optimum conditions.

The highest *T*_*g*_ point refers to OSHEP‐GA (76.49°C) compared to OSHEP‐MD (55.49°C, control) and OSHEP‐WPC (48.12°C). The product may be shelf stable when stored below the *T*_*g*_, as deterioration due to microbial growth and chemical reaction is greatly reduced (Sablani et al., 2007). Therefore, it can be concluded that all OSHEPs produced under optimal conditions are stable at room temperature, and that OSHEP‐GA has better storage stability than OSHEP‐WPC.

### 3.6 Sensory evaluation

The sensory attributes of OSHEPs produced with different carriers, at optimal conditions, are shown in Table 3. In the color and overall acceptance scores, OSHEP‐MD scored the highest, followed by OSHEP‐GA and OSHEP‐WPC. The sweetness scores of OSHEP‐MD and OSHEP‐GA were significantly higher than those of OSHEP‐WPC. The solubility and saltiness scores did not differ significantly (*p* < 0.05). Although the WSI difference between OSHEP‐GA and OSHEP‐WPC was 6%, it was considered to be insufficient for panelists to feel the difference. The sourness score of OSHEP‐GA was the highest of all. The onion taste score was significantly lower in OSHEP‐WPC, while the milky taste score was significantly higher. This suggests that the milky taste was dominant to the onion taste in OSHEP‐WPC, which is logical because WPC is a milk‐based product.

**TABLE 3:** Sensory properties of OSHEPs produced with different carriers

Parameter	Maltodextrin 20 DE	Gum arabic	Whey protein concentrate
Color	5.2 ± 0.2^c^ ^1^	4.6 ± 0.2^b^	4.2 ± 0.3^a^
Solubility	4.2 ± 0.3^a^ ^2^	4.3 ± 0.3^a^	4.5 ± 0.3^a^
Sweetness	5.2 ± 0.2^b^	5.0 ± 0.3^b^	3.7 ± 0.3^a^
Saltiness	2.8 ± 0.3^a^	3.0 ± 0.3^a^	2.6 ± 0.3^a^
Sourness	2.5 ± 0.3^a^	3.5 ± 0.3^b^	2.4 ± 0.3^a^
Onion taste	5.9 ± 0.2^b^	5.7 ± 0.2^b^	4.0 ± 0.3^a^
Milky taste	2.7 ± 0.4^a^	2.9 ± 0.4^a^	5.2 ± 0.3^b^
Overall acceptability	5.1 ± 0.4^c^	4.3 ± 0.3^b^	3.4 ± 0.4^a^

^1^ Values are mean ± standard deviation (of three replicate measurements).

^2^ Means with the same superscript within the same column are not significantly different (*p* < 0.05).

Figure 2C shows the principal component analysis (PCA) biplot for the sensory attributes of OSHEPs produced with different carriers at optimal conditions. The differences and similarities between OSHEPs with respect to their sensory attributes are shown in the PCA. Analytical variables explained 100% of the variability for the first two components of the PCA scope plot, PC1 (80.4%) and PC2 (19.6%). OSHEP‐GA was positively correlated with color, sweetness, onion taste, saltiness, sourness, and overall acceptability, and OSHEP‐WPC exhibited a negative correlation with milky taste and solubility based on the loading plot for Dim1 in Figure 2C. Based on the PCA results, the sensory attributes of OSHEP‐GA and OSHEP‐WPC were found to be distinctive. OSHEP‐GA was correlated with sweetness and overall acceptability, and had high scores for the same, compared with OSHEP‐WPC. In addition, the sweetness score of OSHEP‐GA was similar to that of OSHEP‐MD (generally considered the best carrier in spray drying). Based on sensory evaluation results, it can be concluded that OSHEP‐GA has higher consumer sensory scores than OSHEP‐WPC.

## 4 CONCLUSION

Onion–stevia leaf hot extract powder was manufactured for prospective use as a sweetener, using a spray dryer. GA and WPC were both found to be adequate carriers for the process. The optimal inlet air temperatures and carrier concentrations for manufacturing OSHEP‐GA and OSHEP‐WPC were 148.81 and 144.62°C, and 11.58% and 12.03%, respectively. Physicochemical properties, such as drying yield, color, bulk density, moisture content, water solubility index, particle size, and morphology, were significantly different with different carriers. The drying yield increased with inlet air temperature, up to 170°C, and then decreased slightly, and the highest drying yield was 66.81% at inlet air temperature (150°C) and 10% GA concentration (w/v). The L* value showed a negative correlation with inlet air temperature and a positive correlation with carrier concentration. WSI increased with the concentration of carrier [from 5% to 10% (w/v)] and with inlet air temperature. The results also revealed that the inlet air temperature and carrier concentration had a significant influence on the physicochemical properties and sensory attributes of OSHEP. OSHEP‐WPC has a higher WSI and moisture content than OSHEP‐GA. However, OSHEP‐GA is more stable in powder form, without any holes in the particles, and with better sweetness and overall acceptability of the sensory properties, compared to OSHEP‐WPC. OSHEP‐GA also has a higher *T*_*g*_ value than the positive control (OSHEP‐MD). We conclude that an inlet air temperature of 148.81°C, using GA as a carrier at a concentration of 11.58% (w/v), was the optimal manufacturing condition for the production of a natural sweetener, OSHEP. Specific compounds that should be extracted and quantified in the future include onionin, cysteine sulfoxides, phenolic compounds (rutin, quercetin, and quercetin glucosides), rebaudioside‐A (Reb‐A, B, C, & D), as well as ferulic acid.

## CONFLICT OF INTEREST

Authors have no potential competing or conflict of interest to declare.

## Data Availability

The corresponding author can provide data to support the results of this inquiry upon request.
